# A nomogram for predicting pathological complete response in patients with human epidermal growth factor receptor 2 negative breast cancer

**DOI:** 10.1186/s12885-016-2652-z

**Published:** 2016-08-05

**Authors:** Xi Jin, Yi-Zhou Jiang, Sheng Chen, Ke-Da Yu, Ding Ma, Wei Sun, Zhi-Min Shao, Gen-Hong Di

**Affiliations:** 1 Department of Breast Surgery, Fudan University Shanghai Cancer Center, Shanghai, 200032 China; 2 Department of Oncology, Shanghai Medical College, Fudan University, Shanghai, 200032 China

**Keywords:** HER2 negative breast cancer, Neoadjuvant chemotherapy, Nomogram, Pathological complete response

## Abstract

**Background:**

The response to neoadjuvant chemotherapy has been proven to predict long-term clinical benefits for patients. Our research is to construct a nomogram to predict pathological complete response of human epidermal growth factor receptor 2 negative breast cancer patients.

**Methods:**

We enrolled 815 patients who received neoadjuvant chemotherapy from 2003 to 2015 and divided them into a training set and a validation set. Univariate logistic regression was performed to screen for predictors and construct the nomogram; multivariate logistic regression was performed to identify independent predictors.

**Results:**

After performing the univariate logistic regression analysis in the training set, tumor size, hormone receptor status, regimens of neoadjuvant chemotherapy and cycles of neoadjuvant chemotherapy were the final predictors for the construction of the nomogram. The multivariate logistic regression analysis demonstrated that T4 status, hormone receptor status and receiving regimen of paclitaxel and carboplatin were independent predictors of pathological complete response. The area under the receiver operating characteristic curve of the training set and the validation set was 0.779 and 0.701, respectively.

**Conclusions:**

We constructed and validated a nomogram to predict pathological complete response in human epidermal growth factor receptor 2 negative breast cancer patients. We also identified tumor size, hormone receptor status and paclitaxel and carboplatin regimen as independent predictors of pathological complete response.

**Electronic supplementary material:**

The online version of this article (doi:10.1186/s12885-016-2652-z) contains supplementary material, which is available to authorized users.

## Background

Breast cancer is the most common malignant disease and the second most common cause of cancer death in women [1]. Neoadjuvant chemotherapy has several advantages compared with adjuvant chemotherapy [2]. It increases the rate of breast conservation and offers the opportunity for patients with locally advanced breast cancer to receive surgery. Moreover, sensitivity to different chemotherapy regimens can be assessed, thus helping to make decisions for subsequent treatment.

Pathological complete response (pCR) has been confirmed to predict long-term clinical benefit for patients receiving neoadjuvant chemotherapy and can serve as a dependable endpoint when investigating the efficiency of different treatment regimens [3]. With the application of human epidermal growth factor receptor 2 blockade using neoadjuvant treatments such as trastuzumab, pertuzumab and lapatinib in human epidermal growth factor receptor 2 (HER2) positive patients, the pCR rate of HER2 positive patients is high (16.8–66.2 %) [4]. However, the pCR rate of HER2 negative patients is relatively low (7.0–16.2 % for hormone receptor positive, HER2 negative patients and 33.6–35.0 % for triple negative patients) [3, 5]. Thus, predicting the response to neoadjuvant chemotherapy for HER2 negative patients is essential to optimizing the treatment for individual patients.

Anthracyclines used to be the most common chemotherapeutic agents for breast cancer [6]. However, as taxane-based [7] or platinum-based [8, 9] regimens showed their advantages, the use of anthracyclines has been declining in recent years [10]. The potential impact of this change is still unknown.

A nomogram is a simple graphical representation of a prediction model that helps oncologists assess the predictive information of individual patients [11]. Several earlier studies constructed nomograms to illustrate the impact of different variables on pCR probability [12–14], but none of them focused on HER2 negative patients and different neoadjuvant chemotherapy regimens.

Our current study aims to construct and validate a well-fitting nomogram based on multivariate logistic regression to evaluate the impact of different neoadjuvant chemotherapy regimens as well as the impact of several other variables on the pCR rate among HER2 negative patients in a prospective cohort.

## Methods

### Patient population

Relevant clinical data (age, menopausal status, tumor size, nodal status, regimens of chemotherapy and cycles of chemotherapy), core needle biopsy samples and surgical specimens were collected from Fudan University Shanghai Cancer Center between January 1, 2003 and April 31, 2015.

Overall, 1244 patients who were diagnosed with primary breast cancer and who received neoadjuvant chemotherapy followed by standard surgery were enrolled.

Patients with HER2 positive core needle biopsy samples, with metastatic disease, with missing data or with previous endocrine therapy were not eligible for this study. In total, 429 patients who had missing relevant information, who were HER2 positive or who had received neoadjuvant chemotherapy regimens other than cyclophosphamide, epirubicin and 5-fluorouracil, cyclophosphamide, epirubicin and 5-fluorouracil followed by paclitaxel or docetaxel and epirubicin, navelbine and epirubicin or paclitaxel and carboplatin or paclitaxel and cisplatin were excluded from our study.

**Fig. 1:**
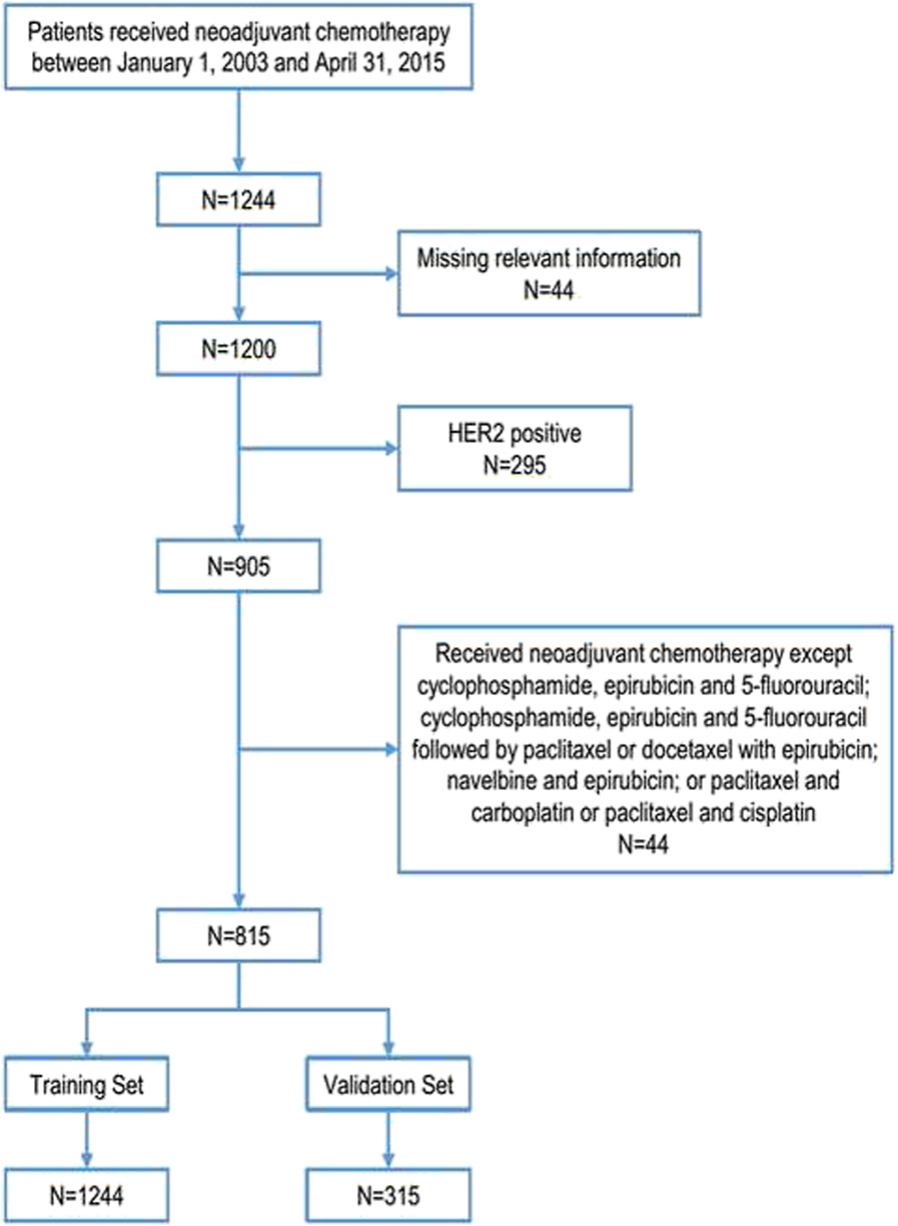
Flow diagram of the study design. A total of 815 Human Epidermal Growth Factor Receptor 2 (HER2) negative patients who received neoadjuvant chemotherapy with the regimen of cyclophosphamide, epirubicin and 5-fluorouracil; cyclophosphamide, epirubicin and 5-fluorouracil followed by paclitaxel or docetaxel and epirubicin; navelbine and epirubicin; or paclitaxel and carboplatin or paclitaxel and cisplatin were included in this study

The remaining 815 patients were randomized into a training set (*N* = 500, enrolled in the nomogram construction) or a validation set (*N* = 315, enrolled in the nomogram external validation) (Fig. 1).

### Pathology and treatment

Estrogen receptor, progestogen receptor status and HER2 status were determined by immunohistochemical analysis, which was performed with formalin-fixed, paraffin-embedded tissue sections using standard protocols for core needle biopsy specimens by the pathology department of Fudan University Shanghai Cancer Center. The cut-off value for estrogen receptor positivity and progestogen receptor positivity was set at 1 %. Absence of both estrogen receptor and progestogen receptor was defined as hormone receptor negative (estrogen receptor negative and progestogen receptor negative); presence of either was defined as hormone receptor positive (estrogen receptor positive or progestogen receptor positive). HER2-positivity was defined as 3 (+) by immunohistochemical or amplification and was confirmed by fluorescence in situ hybridization. Each specimen was examined independently by two experienced pathologists.

The patients in our cohort received one of the following neoadjuvant chemotherapy regimens for a median of 4 cycles (range, 1–6 cycles): navelbine and epirubicin, cyclophosphamide, epirubicin and 5-fluorouracil, paclitaxel with carboplatin/paclitaxel with cisplatin or epirubicin and 5-fluorouracil followed by paclitaxel or docetaxel and epirubicin. pCR was defined as complete disappearance of invasive carcinoma in the breast and regional lymph nodes [3].

### Construction of the nomogram

To develop a well-calibrated and useful nomogram for predicting pCR, possible predictive variables were identified by univariate logistic regression (*P* < 0.05 in univariate logistic regression analysis). The Hosmer-Lemeshow test was used to assess the fitness of the nomogram (*P* > 0.05 indicating good fit) [15]. Multivariate logistic regression analysis was performed to screen independent variables predicting pCR. Odds ratios and 95 % confidence intervals (CI) were calculated.

### Evaluating model performance

The internal validation of our model was performed by a calibration method and the area under the receiver operating characteristic (ROC) curve (AUC). Calibration [16] (visualized as the calibration plot) with a bootstrapping method [17] was used to illustrate the association between the actual probability and the predicted probability. The external validation was achieved by performing the ROC as well as the AUC in a separated population. The AUC ranged from 0 to 1, with the value of 1 indicating perfect concordance, 0.5 indicating no better than chance, and 0 indicating discordance. Statistical differences between different AUCs were investigated by the DeLong method [18].

### Statistical analysis

Chi-square test was used to evaluate the relationship between neoadjuvant chemotherapy regimens and other characteristics. Fisher’s exact test was performed when necessary. All reported *P*-values are two-sided. The statistical analysis was carried out using SPSS (version 20.0; SPSS Company, Chicago, IL) and R software version 3.13 (http://www.r-project.org). The R package with rms, pROC, Hmisc and ggplot2 (available at URL: http://cran.r-project.org/web/packages/) was used (last accessed on March 9, 2015). All relevant R code were shown in Additional file 1.

## Results

### Patient characteristics

Of the 815 HER2 negative patients enrolled in this study, 111 (13.6 %) reached pCR (Table 1). Young patients (≤40 years) [19] had higher pCR rates than older patients (>40 years) (17.0 % versus 12.8 %). Pre-menopausal patients (14.2 %) had higher pCR rates than those who were post-menopausal (12.8 %). Patients with smaller tumor size and more positive lymph nodes reached pCR more easily. hormone receptor negative patients (23.0 %) had higher pCR rates than hormone receptor positive ones (9.8 %). Patients who received the paclitaxel with carboplatin/paclitaxel with cisplatin regimen had higher pCR rates than those who received the cyclophosphamide, epirubicin and 5-fluorouracil, epirubicin and 5-fluorouracil followed by paclitaxel or docetaxel and epirubicin or navelbine and epirubicin regimens (19.4 % versus 1.9 %, 7.8 and 9.8 %, respectively). Patients who received 3 to 4 cycles of neoadjuvant chemotherapy had higher pCR rates (16.1 %) than other subjects. These results were similar in the training and validation sets.

**Table 1:** Clinicopathologic characteristics of patients

	Overall			Training set			Validation set		
	ALL (N)	pCR (N)	pCR rate	ALL (N)	pCR (N)	pCR rate	ALL (N)	pCR (N)	pCR rate
Total	815	111	13.6 %	500	68	13.6 %	315	43	13.7 %
Age									
≤40 years	165	28	17.0 %	105	17	16.2 %	60	11	18.3 %
>40 years	650	83	12.8 %	395	51	12.9 %	255	32	12.5 %
Menopausal status									
Pre-menopausal	457	65	14.2 %	276	40	14.5 %	181	25	13.8 %
Post-menopausal	358	46	12.8 %	224	28	12.5 %	134	18	13.4 %
Tumor size									
T1	89	21	23.6 %	60	15	25.0 %	29	6	20.7 %
T2	346	47	13.6 %	210	30	14.3 %	136	17	12.5 %
T3	235	28	11.9 %	137	15	10.9 %	98	13	13.3 %
T4	145	15	10.3 %	93	8	8.6 %	52	7	13.5 %
Nodal status									
N0	170	22	12.9 %	100	15	15.0 %	70	7	10.0 %
N1	593	79	13.3 %	363	45	12.4 %	230	34	14.8 %
N2	23	4	17.4 %	16	3	18.8 %	7	1	14.3 %
N3	29	6	20.7 %	21	5	23.8 %	8	1	12.5 %
Hormone receptor status									
Negative	235	54	23.0 %	147	36	24.5 %	88	18	20.5 %
Positive	580	57	9.8 %	353	32	9.1 %	227	25	11.0 %
Regimens									
Cyclophosphamide, epirubicin and 5-fluorouracil	107	2	1.9 %	66	1	1.5 %	41	1	2.4 %
Cyclophosphamide, epirubicin and 5-fluorouracil followed by paclitaxel or docetaxel and epirubicin	116	9	7.8 %	73	5	6.8 %	43	4	9.3 %
Navelbine and epirubicin	153	15	9.8 %	94	8	8.5 %	59	7	11.9 %
Paclitaxel and carboplatin or paclitaxel and cisplatin	439	85	19.4 %	267	54	20.2 %	172	31	18.0 %
Cycles									
1-2	97	3	3.1 %	61	2	3.3 %	36	1	2.8 %
3-4	578	93	16.1 %	359	58	16.2 %	219	35	16.0 %
5-6	140	15	10.7 %	80	8	10.0 %	60	7	11.7 %

*Abbreviations*: *pCR* pathological complete response

### Predictors for pCR

In the training set, univariate logistic regression was performed to analyze the association between response to chemotherapy and patient age, menopausal status, tumor size, nodal status, hormone receptor status, regimens of chemotherapy and cycles of chemotherapy (Table 2). Tumor size (*P* = 0.029), hormone receptor status (<0.001), and neoadjuvant chemotherapy regimens (*P* < 0.001) and cycles (*P* = 0.029) were identified to be statistically significant predictors of pCR. No significant differences in pCR rate were observed among patients with different ages, menopausal statuses or nodal statuses.

**Table 2:** Univariate logistic regression analysis of different variables predicting pCR in the training set

	P	OR	95 % CI
Total			
Age	0.385		
≤40 years		1	
>40 years	0.385	0.767	0.423-1.394
Menopausal status	0.518		
Pre-menopausal		1	
Post-menopausal	0.518	0.843	0.502-1.416
Tumor Size	0.029		
T1		1	
T2	0.052	0.500	0.248-1.007
T3	0.014	0.369	0.167-0.815
T4	0.008	0.282	0.111-0.716
Nodal status	0.432		
N0		1	
N1	0.493	0.802	0.426-1.508
N2	0.701	1.308	0.332-5.147
N3	0.328	1.171	0.564-5.561
Hormone receptor status	<0.001		
Negative		1	
Positive	<0.001	0.307	0.182-0.518
Regimens	<0.001		
Cyclophosphamide, epirubicin and 5-fluorouracil		1	
Cyclophosphamide, epirubicin and 5-fluorouracil followed by paclitaxel or docetaxel and epirubicin	0.158	4.779	0.544-42.018
Navelbine and epirubicin	0.094	6.047	0.738-49.558
Paclitaxel and carboplatin or paclitaxel and cisplatin	0.006	16.479	2.236-121.451
Cycles	0.029		
3-4		1	
1-2	0.018	0.176	0.042-0.740
5-6	0.143	0.577	0.264-1.261

*Abbreviations*: *pCR* pathological complete response, *OR* odds ratio, *CI* confidence interval

Given that the baseline patient characteristics of different neoadjuvant chemotherapy regimens were not in concordance (Additional file 2), we performed multivariate logistic regression analysis to screen for the independent predictors of pCR (Table 3). Relative to T1 patients, T4 patients were less likely to achieve pCR [*P* = 0.015, odds ratio =0.281 (95 % CI: 0.101–0779)]. The odds ratio of hormone receptor positive patients was 0.224 (95 % CI: 0.125–0.400); for hormone receptor negative patients, it was 1 (*P* < 0.001). After adjustment for tumor size, hormone receptor status and neoadjuvant chemotherapy cycles, those who received paclitaxel with carboplatin/paclitaxel with cisplatin had a statistically significant higher rate of pCR Compared with patients who received cyclophosphamide, epirubicin and 5-fluorouracil [*P* = 0.003, odds ratio =27.696 (95 % CI: 3.131–245.030)]. Patients who received epirubicin and 5-fluorouracil followed by paclitaxel or docetaxel and epirubicin, navelbine and epirubicin had higher odds ratio than those who received cyclophosphamide, epirubicin and 5-fluorouracil (6.973 and 4.701 versus 1), but the difference was not statistically significant. Although we found out the trends that patients receiving only 1–2 cycles neoadjuvant chemotherapy showed lower probability for pCR (odds ratio: 0.579) while patients receiving 5–6 cycles neoadjuvant chemotherapy showed higher probability for pCR (odds ratio: 2.338) than those who received 3–4 cycles of neoadjuvant chemotherapy, different neoadjuvant chemotherapy cycles were not statistically significant for predicting pCR.

**Table 3:** Multivariable logistic regression analysis of possible variables (*P*<0.05 in univariate logistic regression analysis) predicting pCR

	P	OR	95 % CI
Tumor size			
T1		1	
T2	0.186	0.576	0.255-1.304
T3	0.544	0.737	0.275-1.975
T4	0.015	0.281	0.101-0.779
Hormone receptor status			
Negative		1	
Positive	<0.001	0.224	0.125-0.400
Regimens			
Cyclophosphamide, epirubicin and 5-fluorouracil		1	
Cyclophosphamide, epirubicin and 5-fluorouracil followed by paclitaxel or docetaxel and epirubicin	0.208	4.673	0.423-51.590
Navelbine and epirubicin	0.078	6.999	0.804-60.897
Paclitaxel and carboplatin or paclitaxel and cisplatin	0.003	27.696	3.131-245.030
Cycles			
3-4		1	
1-2	0.500	0.579	0.118-2.834
5-6	0.217	2.338	0.606-9.017

*Abbreviations*: *pCR* pathological complete response, *OR* odds ratio, *CI* confidence interval

We performed logistic regression to explore the predictors for pCR separately both in hormone receptor positive and negative cohort. Tumor status (T3 vs T1, T4 vs T1) was only statistically significant in hormone receptor positive patients and not in hormone receptor negative patients. Nodal status was not statistically significant in either group. Epirubicin and 5-fluorouracil followed by paclitaxel or docetaxel with epirubicin and navelbine with epirubicin showed statistically significant superiority to cyclophosphamide, epirubicin and 5-fluorouracil regimens in hormone receptor negative patients, but not in hormone receptor positive patients, while paclitaxel with carboplatin/paclitaxel with cisplatin regimen treated patients had statistically significant higher pCR in overall patients. Only hormone receptor negative patients who received 1–2 cycles had statistically significant lower pCR rate than those receiving 3–4 cycles (Additional file 3). In addition, we found that among paclitaxel with carboplatin/paclitaxel with cisplatin treated patients, hormone receptor negative (triple negative) patients had higher rate of pCR rate (38.9 %, Chi-square test *P* < 0.001) than hormone receptor positive patients (13.0 %) (Additional file 4).

### Construction and validation of the nomogram

**Fig. 2:**
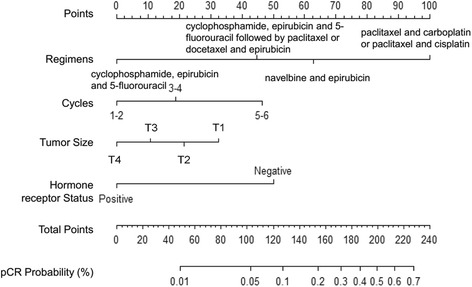
Nomogram predicting the probability of pathological complete response (pCR) after neoadjuvant chemotherapywith the regimen of cyclophosphamide, epirubicin and 5-fluorouracil; cyclophosphamide, epirubicin and 5-fluorouracil followed by paclitaxel or docetaxel and epirubicin; navelbine and epirubicin; or paclitaxel and carboplatin or paclitaxel and cisplatin

Statistically significant predictors in univariate logistic regression analysis (tumor size, hormone receptor status, neoadjuvant chemotherapy regimens and cycles) were included into the nomogram construction (Fig. 2). The total points were added up by the points of each variable (top scale). The pCR probability depended on the total points (bottom scale). The *P*-value for the Hosmer-Lemeshow test was 0.817, indicating good fit of the model.

**Fig. 3:**
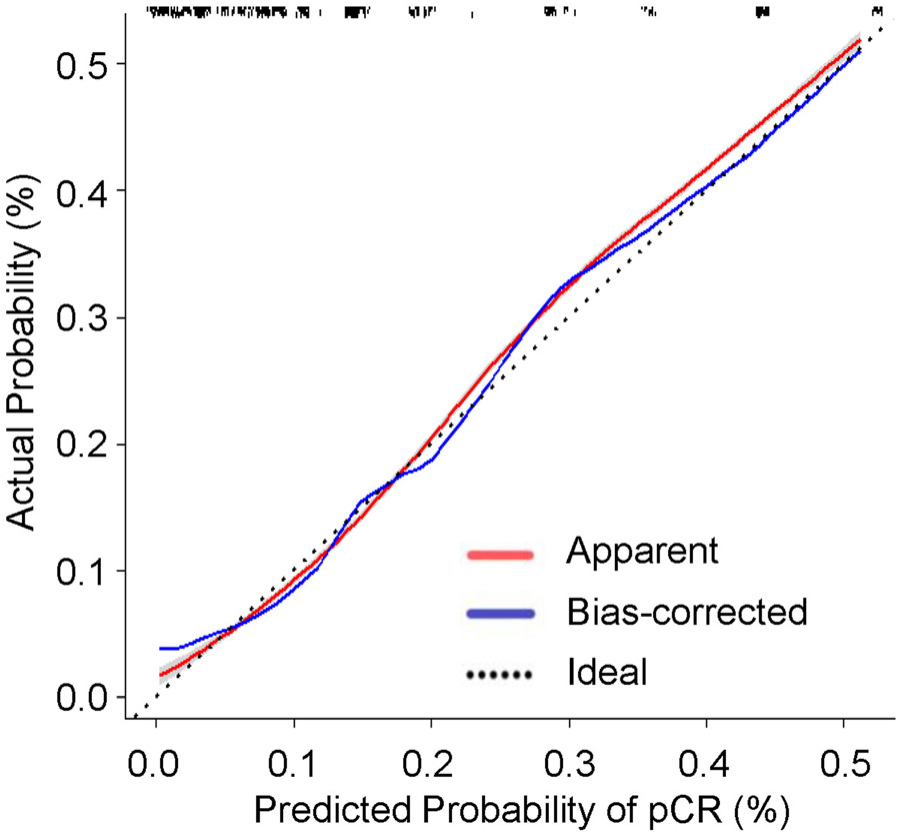
Calibration plot of the nomogram for the probability of pathological complete response (pCR) (bootstrap 1000 repetitions)

The calibration of the nomogram was performed internally by a calibration plot with bootstrap sampling (*n* = 1000) (Fig. 3). The calibration plot illustrated that the nomogram was well calibrated.

**Fig. 4:**
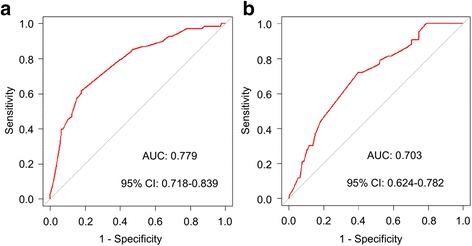
Validation of the Nomogram. **a** Internal validation using receiver operating characteristic (ROC) curve. The area under the ROC curve (AUC) is 0.779, 95 % confidence intervals (CI): 0.718–0.839. **b** External validation using ROC. The AUC is 0.703, 95 % CI: 0.622–0.780

Next, we constructed the ROC to further validate the nomogram internally in the training set (Fig. 4a) and externally in the validation set (Fig. 4b). In the training set, the AUC was 0.779 (95 % CI: 0.718–0.839). In the validation set, the AUC was slightly lower: 0.703 (95 % CI: 0.624–0.782). The difference between two AUCs was not statistical significant (*P* = 0.132). These results illustrated that the predicted and observed pCR probabilities were concordant.

### Nomogram performance in individual patients

To display the application of the nomogram, we took two breast cancer patients who had received neoadjuvant chemotherapy as examples. The first patient was to receive epirubicin and 5-fluorouracil followed by paclitaxel or docetaxel and epirubicin as an neoadjuvant chemotherapy regimen (45 points) for four cycles (19 points); his tumor size was T2 (22 points) and his hormone receptor status was positive (0 points). According to the nomogram, his probability of reaching pCR was approximately 0.01 to 0.05 (total points: 86). The second patient was to receive paclitaxel with carboplatin/paclitaxel with cisplatin as an neoadjuvant chemotherapy regimen (100 points) for four cycles (19 points); his tumor size was T4 (0 points) and his hormone receptor status was negative (50 points). According to the nomogram, his probability of reaching pCR was approximately 0.2 to 0.3 (total points: 169). As a result of using this nomogram, clinicians can obtain an overview of the response of different treatments for individual patients.

## Discussion

Based on the logistic regression, we screened for predictors and constructed a concise and well fitted nomogram containing the variables of tumor size, hormone receptor status, regimens of neoadjuvant chemotherapy and cycles of neoadjuvant chemotherapy to predict the pCR rate of HER2 negative patients. This would be a convenient application for clinicians. Using the method of calibration plot with bootstrap sampling, as well as internal and external validation by AUC and ROC, the nomogram proved to be of good fitness.

In this study, we first screened variables that could predict the response to neoadjuvant chemotherapy by univariate logistic regression. Tumor size, hormone receptor status, and neoadjuvant chemotherapy regimens and cycles were included in the construction of the nomogram. Next, we intended to identify several independent predictors of the pCR rate. In the multivariate logistic regression analysis, we found that T4 status (*P* = 0.015, odds ratio: 0.281, 95 % CI: 0.101–0.779), hormone receptor positivity (*P* < 0.001, odds ratio: 0.224, 95 % CI: 0.125–0.400) and receiving the paclitaxel with carboplatin/paclitaxel with cisplatin regimen (*P* = 0.003, odds ratio: 27.696, 95 % CI: 3.131–245.030) were the most important predictors of pCR in this model. Compared with T1 patients, T4 patients had worse responses to chemotherapy, which is consistent with previous research [20]. Hormone receptor status was another independent predictor, and hormone receptor positive patients had lower pCR rates than hormone receptor negative patients. Our findings are concordant with previous studies [20–22] that show that hormone receptor positive tumor cells are less sensitive to chemotherapy compared with hormone receptor negative cells. Patients treated with paclitaxel with carboplatin/paclitaxel with cisplatin had better neoadjuvant chemotherapy responses compared with those treated with cyclophosphamide, epirubicin and 5-fluorouracil. Anthracyclines such as epirubicin and doxorubicin were once considered to be the most effective agents in the treatment of breast cancer, but the use of them has been declining recently [10]. In our current study, the anthracycline-based regimens included cyclophosphamide, epirubicin and 5-fluorouracil, epirubicin and 5-fluorouracil followed by paclitaxel or docetaxel with epirubicin and navelbine with epirubicin. Cyclophosphamide, epirubicin and 5-fluorouracil was the standard anthracycline-based regimen, and the pCR rate after 6 cycles of cyclophosphamide, epirubicin and 5-fluorouracil was reported to be 14–15 % [23, 24]. However, only 1.9 % of patients who received cyclophosphamide, epirubicin and 5-fluorouracil in our study reached pCR, which may be partially due to the relatively higher proportion of larger tumor size (T3: 50.5 %; T4: 13.1 %) and fewer neoadjuvant chemotherapy cycles received (1–2 cycles: 49.5 %) in the cyclophosphamide, epirubicin and 5-fluorouracil cohort. The total pCR rate for epirubicin and 5-fluorouracil followed by paclitaxel or docetaxel and epirubicin patients was low (7.8 %) which may due to the relatively higher proportion of hormone receptor positive patients (87.1 %). The cumulative cardiac toxicity of anthracyclines has also limited its use, especially in older patients or in those with cardiovascular comorbidities. Therefore, non-anthracycline based regimens are required. Paclitaxel, a mitotic inhibitor and anti-microtubule agent, results in a G2-M phase arrest [25]. Carboplatin and cisplatin share similar anti-cancer mechanisms, as they are both DNA alkylating agents [26]. The combination of paclitaxel and platinum is now widely used in breast cancer patients, and the agents have no overlapping toxicities [27]. Previous research has already assessed the efficacy and the toxicity of the paclitaxel with carboplatin/paclitaxel with cisplatin regimen in adjuvant therapy and in neoadjuvant chemotherapy. The pCR rate of patients who received paclitaxel with carboplatin/paclitaxel with cisplatin as neoadjuvant chemotherapy ranged from 9.5 to 19.4 % [28, 29]. The data from our center is 19.4 %, similar to previous studies. The paclitaxel with carboplatin/paclitaxel with cisplatin regimen achieved greater therapeutic effect than any anthracycline-based regimens, especially in triple negative breast cancer patients. Triple negative breast cancer patients have higher rate of BRCA1/2 (Breast Cancer 1/2) mutation and are sensitive to platinum (because of the deficiencies in the DNA repair mechanism) [30, 31]. In aggregate, these results suggested that platinum contained therapy is recommend for triple negative breast cancer patients.

The nomogram provides a simple graphical representation of sophisticated statistical prediction models and has been accepted as a reliable tool for predicting clinical events. It is especially widely used in oncology [11]. Previously, several studies constructed nomograms to predict the pCR rate of neoadjuvant chemotherapy. The first of these studies appeared in 2005 [12]. Rouzier et al. constructed two nomograms to predict the responses to anthracycline-based neoadjuvant chemotherapy and to combined anthracycline and paclitaxel neoadjuvant chemotherapy. The nomograms were validated externally. Colleoni et al. constructed a nomogram to predict pCR probability based on a population of 783 patients [13]. The nomogram proved to be well fitted after external validation by 101 patients. However, the HER2 status was not mentioned in these two studies. Keam et al. constructed another nomogram to predict pCR and predict which patients would not relapse [14]. Overall, 370 patients who received 3 cycles of neoadjuvant docetaxel or doxorubicin were included in this study. However, the HER2 status was not stratified and the validation of the nomogram was only performed internally. The advantage of our research is that we first constructed a nomogram for predicting the pCR rate among HER2 negative patients, and the nomogram was proven to be well fitted by internal and external validation. We selected HER2 negative patients as our target population for two reasons. First, the pCR rates of these patients were relatively low, so individualized therapy for each patient was required. Second, confounding variables such as HER2 blockade treatment were limited in our cohort. Additionally, we discovered that paclitaxel with carboplatin/paclitaxel with cisplatin was the more favored neoadjuvant chemotherapy regimen compared with cyclophosphamide, epirubicin and 5-fluorouracil in HER2 negative patients.

One limitation of our study was that the design was a single center analysis. Applying the nomogram in another database will greatly improve the power of our current result, and we have carefully searched through existing public databases. Unfortunately, we were unable to find a proper database containing all of the variables analyzed in our current study (age, menopause status, tumor size, nodal status, hormone receptor status, neoadjuvant chemotherapy regimens, neoadjuvant chemotherapy cycles and response to neoadjuvant chemotherapy). We expect to assess the nomogram with large-scale randomized prospective clinical trials. The efficacy and safety of the paclitaxel with carboplatin/paclitaxel with cisplatin regimen used in neoadjuvant chemotherapy also needs to be assessed. Another limitation was that the molecular mechanisms of the paclitaxel with carboplatin/paclitaxel with cisplatin regimen (more so than the cyclophosphamide, epirubicin and 5-fluorouracil regimen) were unclear so further research is required in the future to study these mechanisms.

## Conclusion

Our current study screened for several predictors and constructed a well fitted nomogram based on those predictors to predict the pCR rate among HER2 negative breast cancer patients.

## Abbreviations

AUC, the area under the ROC curve; CI, confidence interval; HER2, human epidermal growth factor receptor 2; pCR, pathological complete response; ROC, receiver operating characteristic

## Additional files

Additional file 1: R running of Nomogram for predqicting pCR. (DOC 16 kb) Additional file 2: Patient baseline characteristics of different NCT regimens. (DOC 16 kb) Additional file 3: Univariate logistic regression analysis of different variables predicting pathological complete response (pCR) in hormone receptor (HR) positive and negative cohorts. CEF: cyclophosphamide, epirubicin and 5-fluorouracil; CI: Confidence interval; E + P: cyclophosphamide, epirubicin and 5-fluorouracil followed by paclitaxel or docetaxel and epirubicin; NE: navelbine and epirubicin; OR: odds ratios; PC: paclitaxel and carboplatin or paclitaxel and cisplatin. (DOC 1415 kb) Additional file 4: Pathological complete response (pCR) of different neoadjuvant chemotherapy (NCT) regimens in hormone receptor (HR) positive and negative cohorts. (DOC 152 kb)
