# Supplementary material for: A nomogram for predicting pathological complete response in patients with human epidermal growth factor receptor 2 negative breast cancer
Source: BMC Cancer. 2016 Aug 5;16:606. doi: 10.1186/s12885-016-2652-z (PMC4974800; doi:10.1186/s12885-016-2652-z)
Supplement: Additional file 1: — R running of Nomogram for predqicting pCR. (DOC 16 kb) [file 12885_2016_2652_MOESM1_ESM.doc]

**R running of Nomogram for predqicting pCR**

This is the code of nomogram development and validation using R software. Text in blue represents the relavant output.

**## Nomogram Construction**

F1<-read.csv(file.choose(),header=T)

attach(F1)

ddist<-datadist(Regimens,Cycles,T,HR,)

options(datadist='ddist')

logi<-lrm(pCR~Regimens+Cycles+T+HR,x=TRUE, y=TRUE)

nomo<-nomogram(logi, fun=plogis,

fun.at=c(.001, .01, .05, seq(.1,.9, by=.1), .95, .99, .999),lp=F, funlabel="pCR")

plot(nomo)

| Predictors | | Points |
| --- | --- | --- |
| NCT Regimens | CEF | 0 |
|  | E+P | 47 |
|  | NE | 63 |
|  | PC | 100 |
| NCT Cycles | 1-2 | 0 |
|  | 3-4 | 24 |
|  | 5-6 | 49 |
| Tumor Size | T1 | 33 |
|  | T2 | 22 |
|  | T3 | 11 |
|  | T4 | 0 |
| HR Status | Negative | 51 |
|  | Positive | 0 |

| Total Points | pCR Rate |
| --- | --- |
| 52 | 0.01 |
| 108 | 0.05 |
| 133 | 0.1 |
| 160 | 0.2 |
| 179 | 0.3 |
| 194 | 0.4 |
| 207 | 0.5 |
| 221 | 0.6 |
| 236 | 0.7 |

**##Calibrate curve construction**

cal<-calibrate(logi, method="boot", B=1000, bw=FALSE, rule="p", type="individual", sls=.05, aics=0, force=NULL, estimates=T, pr=FALSE, smoother="lowess", digits=NULL)

plot(cal)

**## ROC of Training set construction**

library(pROC)

F1<-read.csv(file.choose(),header=T)

attach(F1)

logi<-lrm(pCR~Regimens+Cycles+T+HR,x=TRUE, y=TRUE)

Predi<-predict(logi,F1,type="lp")

roc1<-roc(F1$pCR,Predi)

plot(roc1,font=2,legacy.axes=TRUE)

Area under the curve: 0.7794

ci.auc(roc1)

95% CI: 0.7189-0.8398 (DeLong)

**## ROC of Validation set construction**

library(pROC)

F1<-read.csv(file.choose(),header=T)

attach(F1)

logi<-lrm(pCR~Regimens+Cycles+T+HR,x=TRUE, y=TRUE)

F2<-read.csv(file.choose(),header=T)

attach(F2)

Predi<-predict(logi,F2,type="lp")

roc2<-roc(F2$pCR,Predi)

plot(roc2,col=c("red","yellow","green"),font=2,legacy.axes=TRUE)

Area under the curve: 0.703

ci.auc(roc2)

95% CI: 0.6242-0.7818 (DeLong)

**## Compare the AUC of two ROC curves**

roc.test(roc1,roc2)

DeLong's test for two ROC curves

data: roc1 and roc2

D = 1.5077, df = 650.34, p-value = 0.1321

alternative hypothesis: true difference in AUC is not equal to 0

sample estimates:

AUC of roc1 AUC of roc2

0.7793607 0.7029754
