# Supplementary material for: A nomogram for predicting pathological complete response in patients with human epidermal growth factor receptor 2 negative breast cancer
Source: BMC Cancer. 2016 Aug 5;16:606. doi: 10.1186/s12885-016-2652-z (PMC4974800; doi:10.1186/s12885-016-2652-z)
Supplement: Additional file 2: — Patient baseline characteristics of different NCT regimens. (DOC 16 kb) [file 12885_2016_2652_MOESM2_ESM.doc]

**Additional file 2:** Patient baseline characteristics of different NCT regimens.

|  | **NCT Regimens** | | | | | | | | **P** |
| --- | --- | --- | --- | --- | --- | --- | --- | --- | --- |
|  | **CEF (N=107)** | | **E+P (N=116)** | | **NE (N=153)** | | **PC (N=439)** | |  |
|  | **N** | **Rate** | **N** | **Rate** | **N** | **Rate** | **N** | **Rate** |  |
| Tumor Size |  |  |  |  |  |  |  |  | <0.001 |
| T1 | 0 | 0.0% | 39 | 33.6% | 0 | 0.0% | 50 | 11.4% |  |
| T2 | 39 | 36.4% | 35 | 30.2% | 47 | 30.7% | 225 | 51.3% |  |
| T3 | 54 | 50.5% | 17 | 14.7% | 92 | 60.1% | 72 | 16.4% |  |
| T4 | 14 | 13.1% | 25 | 21.6% | 14 | 9.2% | 92 | 21.0% |  |
| HR Status |  |  |  |  |  |  |  |  | <0.001 |
| HR negative | 56 | 52.3% | 15 | 12.9% | 56 | 36.6% | 108 | 24.6% |  |
| HR positive | 51 | 47.7% | 101 | 87.1% | 97 | 63.4% | 331 | 75.4% |  |
| NCT Cycles |  |  |  |  |  |  |  |  | <0.001 |
| 3-4 | 43 | 40.2% | 9 | 7.8% | 103 | 67.3% | 423 | 96.4% |  |
| 1-2 | 53 | 49.5% | 6 | 5.2% | 30 | 19.6% | 8 | 1.8% |  |
| 5-6 | 11 | 10.3% | 101 | 87.1% | 20 | 13.1% | 8 | 1.8% |  |

Abbreviations: HR: hormone receptor; NCT: neoadjuvant chemotherapy; CEF: cyclophosphamide, epirubicin and 5-fluorouracil; E+P: cyclophosphamide, epirubicin and 5-fluorouracil followed by paclitaxel or docetaxel and epirubicin; NE: navelbine and epirubicin; PC: paclitaxel and carboplatin or paclitaxel and cisplatin.
