# Supplementary material for: A nomogram for predicting pathological complete response in patients with human epidermal growth factor receptor 2 negative breast cancer
Source: BMC Cancer. 2016 Aug 5;16:606. doi: 10.1186/s12885-016-2652-z (PMC4974800; doi:10.1186/s12885-016-2652-z)
Supplement: Additional file 3: — Univariate logistic regression analysis of different variables predicting pathological complete response (pCR) in hormone receptor (HR) positive and negative cohorts. CEF: cyclophosphamide, epirubicin and 5-fluorouracil; CI: Confidence interval; E + P: cyclophosphamide, epirubicin and 5-fluorouracil followed by paclitaxel or docetaxel and epirubicin; NE: navelbine and epirubicin; OR: odds ratios; PC: paclitaxel and carboplatin or paclitaxel and cisplatin. (DOC 1415 kb) [file 12885_2016_2652_MOESM3_ESM.doc]

**Additional file 3.** Univariate logistic regression analysis of different variables predicting pathological complete response (pCR) in hormone receptor (HR) positive and negative cohorts. CEF: cyclophosphamide, epirubicin and 5-fluorouracil; CI: Confidence interval; E+P: cyclophosphamide, epirubicin and 5-fluorouracil followed by paclitaxel or docetaxel and epirubicin; NE: navelbine and epirubicin; OR: odds ratios; PC: paclitaxel and carboplatin or paclitaxel and cisplatin.

**
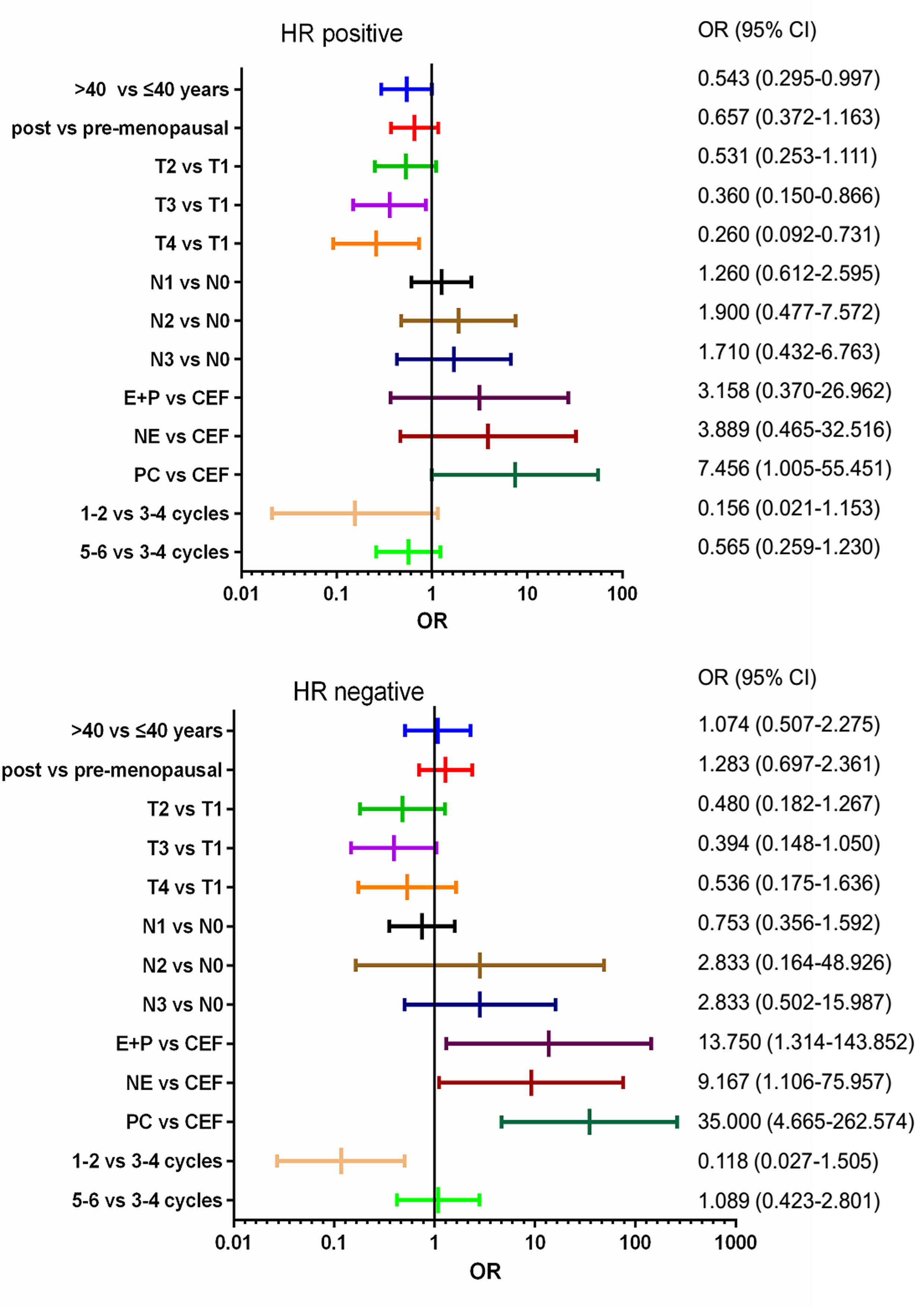
**
