# Supplementary material for: A nomogram for predicting pathological complete response in patients with human epidermal growth factor receptor 2 negative breast cancer
Source: BMC Cancer. 2016 Aug 5;16:606. doi: 10.1186/s12885-016-2652-z (PMC4974800; doi:10.1186/s12885-016-2652-z)
Supplement: Additional file 4: — Pathological complete response (pCR) of different neoadjuvant chemotherapy (NCT) regimens in hormone receptor (HR) positive and negative cohorts. (DOC 152 kb) [file 12885_2016_2652_MOESM4_ESM.doc]

**Additional file 4.** Pathological complete response (pCR) of different neoadjuvant chemotherapy (NCT) regimens in hormone receptor (HR) positive and negative cohorts.

CEF: cyclophosphamide, epirubicin and 5-fluorouracil; E+P: cyclophosphamide, epirubicin and 5-fluorouracil followed by paclitaxel or docetaxel and epirubicin; NE: navelbine and epirubicin; PC: paclitaxel and carboplatin or paclitaxel and cisplatin.

**
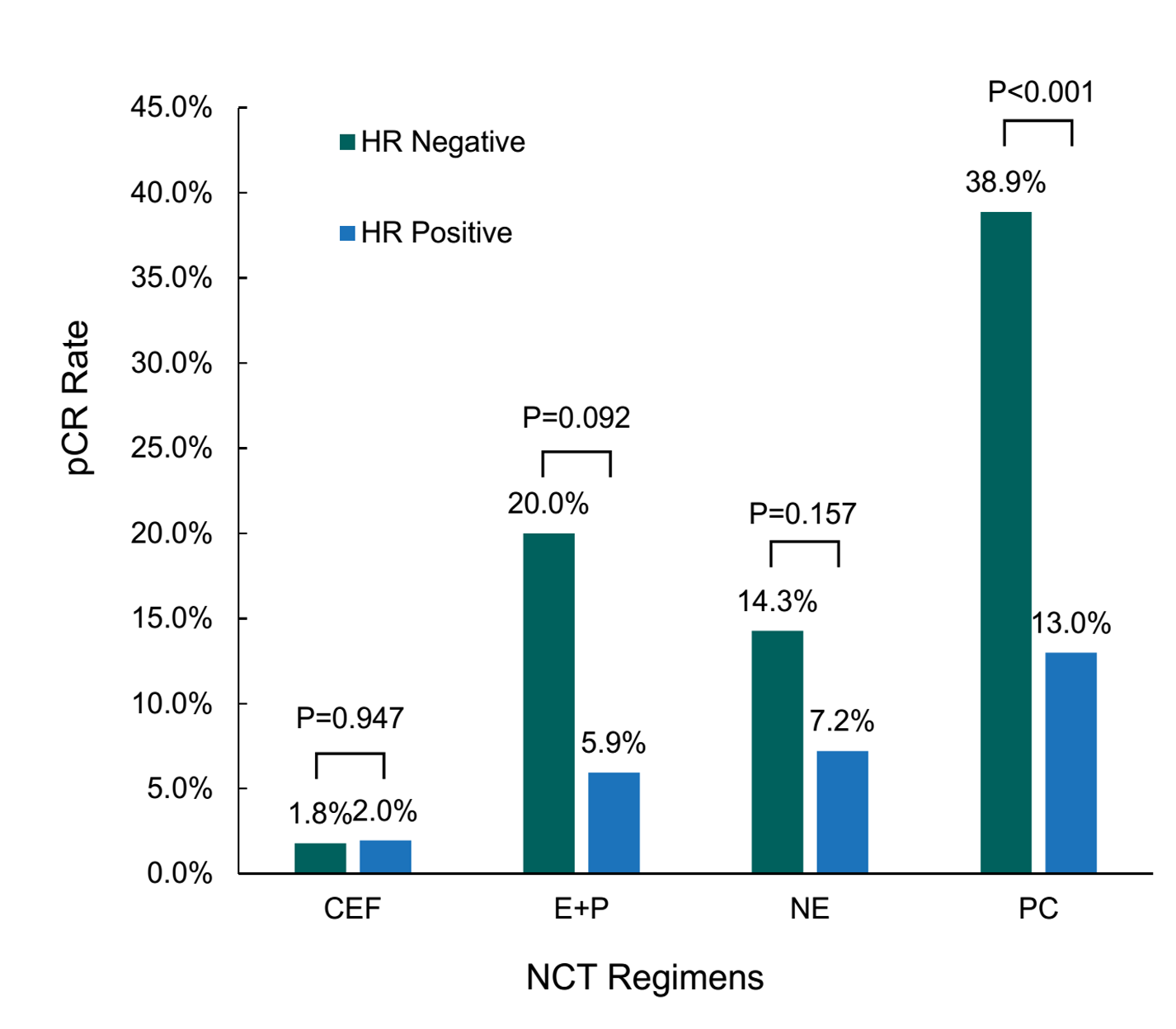
**
